# Cancer

**Published:** 1841-01

**Authors:** 


### Art. VIII.

*Cancer*. By Walter Hayle Walshe, m.d. (*Cyclopædia of Practical Surgery*, Parts VI. & VII.)—*London*, 1840. Royal 8vo, pp. 102.

The great importance of the subject of cancer, and the admirable and elaborate manner in which it is here treated, induce us to deviate from our usual practice of not noticing individual articles in periodical works. With the exception of one or two intentional omissions, to be supplied under the proper alphabetical heads, Dr. Walshe's memoir, indeed, may be considered a complete monography. It is equal in size to many distinct works, and surpasses in merit the majority that come into our hands. It is divided into two parts: in the first of which cancer in general is considered; and in the second, the disease as it occurs in those tissues and organs in which it is likely to come under the notice of the practical surgeon. The first part is that to which our attention must be principally confined. Turning to the end of it for an instant, we extract the definition of cancer, which Dr. Walshe deduces from all that he has said before: "Cancer is a disease anatomically characterized by the presence of scirrhus, encephaloid, or colloid, originating in a general vitiation of the economy, and possessing the properties of assimilation, of reproduction, and of destroying life by a peculiar cachexia." (p. 650.)

Assuming *cancer* or *carcinoma* as a genus, Dr. Walshe recognizes encephaloid, scirrhus and colloid, as species. But, as most writers on the subject have hitherto been more keenly perceptive of the differences of these products, he finds himself under the necessity of insisting on their analogies, as maintained by Young and Carswell, and Laennec, Otto, Cruveilhier, and Müller, in order to justify his grouping them together, and separating them from all other morbid formations. Accordingly, he refers to the points in which they agree *anatomically*, *chemically*, *physiologically*, and *pathologically*; and then remarks that their title to be united is quite as strong in respect of practical medicine and surgery as in respect of scientific pathology; a consideration, it is justly observed, of the highest importance.

In a table, which we subjoin, our author displays the method, according to which, as apparently best in accordance with the present state of knowledge, he treats of carcinoma in general.

| Family. | Class. | Order. | Genus. | Species. | Varieties. | Synonyms of the Species. |
|---|---|---|---|---|---|---|
| Adventitious Formations. | Heterologous Formations. | Tissues. | Cancer, or Carcinoma. | Encephaloid | Common vascular sarcoma. } Abernethy.<br>Mammary sarcoma? } Abernethy.<br>Solanoid. Recamier. Zang.<br>Nephroid. *Idem.*<br>Napiform. *Idem.*<br>Carcinoma fasciculatum vel hyalinum. Müller.<br>Fungus hæmatodes. Hey.<br>Hæmatode cancer. Auct. Gall. | Spongy or ossivorous tumour. Ruysch. Palletta.<br>Struma fungosa (testis). Callisen.<br>Spongoid inflammation. Burns.<br>Milt-like tumour. Munro.<br>Medullary sarcoma. Abernethy.<br>Cerebriform disease, or cancer. Laennec.<br>Pulpy testicle. Baillie.<br>Carcinus spongiosus. Good.<br>Carcinoma spongiosum. Young.<br>Fungoid disease. A. Cooper. Hodgkin.<br>Medullary fungus. Maunoir. Chelius.<br>Acute fungous tumour. C. Bell.<br>Medullary cancer. Travers.<br>Cephaloma. Hooper. Carswell.<br>Carcinoma medullare. Müller.<br>Soft cancer. Auct. Var. |
| | | | | Scirrhus . . | Pancreatic sarcoma? Abernethy.<br>Napiform. } Recamier.<br>Chondroid. } Recamier.<br>Lardaceous tissue. Auct. Gall.<br>Carcinoma reticulare. Müller. | Carcinomatous sarcoma. Abernethy.<br>Carcinoma scirrhosum. Young.<br>Scirrhous cancer. Travers.<br>Scirrhoma. Carswell.<br>Carcinoma simplex vel fibrosum. Müller.<br>Stone cancer. Auct. Var. |
| | | | | Colloid . . | Pultaceous cancer. } Cruveilhier.<br>Pearly alveolar ditto. } Cruveilhier. | Areolar gelatiniform cancer. Cruveilhier.<br>Carcinoma alveolare. Müller.<br>Gum cancer. Hodgkin. |

It will be seen by this table that carcinoma is distinguished as an adventitious heterologous tissue. The circumstance of being a tissue, susceptible of undergoing all the changes of increase and decay, is an important feature of carcinoma, and it is duly dwelt on by Dr. Walshe; but he remarks that Adams, Baron, Carmichael, and Cruveilhier go too far in their views regarding the independent vitality of carcinoma.

Before entering into an examination of the chapters on the Anatomy, Physiology, Pathology, and Therapeia of Carcinoma, we would have the reader accompany us to p. 647, where we find Dr. Walshe exhibiting tabularly the characteristics of the three species of carcinoma; thus

| *Encephaloid.* | *Scirrhus.* | *Colloid.* |
|---|---|---|
| Resembles lobulated cerebral matter. | Resembles rind of bacon traversed by cellulo-fibrous septa. | Has the appearance of particles of jelly inlaid in a regular alveolar bed. |
| Is commonly opaque from its earliest formation. | Has a semitransparent glossiness. | The contained matter is strikingly transparent. |
| Is of dead white colour. | Has a clear whitish or bluish yellow tint. | Greenish yellow is its predominant hue. |
| Contains a multitude of minute vessels. | Is comparatively ill supplied with vessels. | (Its vessels have not been sufficiently examined as yet.) |
| Is less hard and dense than scirrhus. | Is exceedingly firm and dense. | The jelly-like *matter* is exceedingly soft; a colloid *mass* is, however, firm and resisting. |
| Is frequently found in the veins issuing from the diseased mass. | Has not been distinctly detected in this situation. | The pultaceous variety has been detected in the veins. |
| The predominant microscopical elements are globular, not always distinctly cellular, and caudate corpuscula. | The main microscopical constituents are juxtaposed nuclear cells; caudate corpuscula do not exist in it. | Is composed of cells in a state of *emboîtement*. |
| Occasionally attains an enormous bulk. | Rarely acquires larger dimensions than an orange. | Observes a mean in this respect. |
| Has been observed in almost every tissue of the body. | Its seat, as ascertained by observation, is somewhat more limited. | Has so far been seen in a limited number of parts only. |
| Very commonly coexists in several parts or organs of the same subject. | Is not unusually solitary. | Has rarely been met with in more than one organ. |
| Is remarkable for its occasional vast rapidity of growth. | Ordinarily grows slowly. | Grows with a medium degree of rapidity. |
| Is frequently the seat of interstitial hemorrhage and deposition of black or bistre-coloured matter. | Is comparatively rarely the seat of these changes. | |
| When softened into a pulp appears as a dead white or pink opaque matter of creamy consistence. | Resembles, when softened, a yellowish brown semitransparent gelatinous matter. | Undergoes no visible change of the kind. |
| Subcutaneous tumours are slow to contract adhesion with the skin. | Scirrhus thus situated usually becomes adherent. | |
| Ulcerated encephaloid is frequently the seat of hemorrhage, followed by rapid fungous development. | Scirrhous ulcers much less frequently give rise to hemorrhage, and fungous growths (provided they retain the scirrhous character) are now more slowly and less abundantly developed. | |
| The progress of the disease after ulceration is commonly very rapid. | There is not such a remarkable change in the rate of progress of the disease after ulceration has set in. | |
| Is the most common form under which secondary cancer exhibits itself. | | |
| Is the species of cancer most frequently observed in young subjects. | Is much less common before puberty. | Has so far been observed in adults only. |

Great as the number and varieties of these distinctive characters of encephaloid, scirrhus, and colloid are, they are insufficient, Dr. Walshe remarks, to counterbalance the weighty reasons afforded by the following facts for uniting them into a genus, reasons which induce some eminent pathologists to regard them as one and the same formation primarily: 1. The different species are found coexisting in different organs in the same subject. 2. They are even met with in one and the same organ in close proximity. 3. After the ablation of a cancerous tumour, the reproduced growth frequently belongs to a different species from the original: thus, encephaloid follows scirrhus; scirrhus more rarely encephaloid (Müller); encephaloid appears in distant parts after the removal of colloid. 4. In the hard state encephaloid and scirrhus are not to be distinguished by their physical characters. 5. Structure possessing the appearance of scirrhus may soften into true cerebriform pulp.

We now proceed to glance at the mode in which the anatomy, physiology, pathology, and therapeia of carcinoma are treated.

Under the head of Anatomy, Dr. Walshe considers the gross anatomical characters, the microscopical characters, the chemical characters, the form, and the varieties, successively of encephaloid, scirrhus, and colloid. All these points are very ably and fully discussed. A very good abridgment, illustrated by engravings, is given of the microscopical researches of Müller, to which we lately called the attention of our readers on the occasion of their coming before us in an English dress, by the care of Dr. West. This part of Dr. Walshe's memoir, therefore, need not detain us, especially as our limited space will, we fear, be scarcely sufficient for a very brief notice of the important practical matter with which the memoir abounds.

Colloid being the least generally known of the three species of carcinoma, it may be interesting to the reader to have presented to him the following account which Dr. Walshe gives of a specimen of the disease affecting the stomach recently examined by him; some of the characteristics of colloid, in comparison with those of encephaloid and scirrhus, we have already quoted ; we would only further premise that the morbid production in question was first distinguished, described, and named by Laennec. A tolerably accurate description of it was also given in 1816 by Otto; and an illustrative engraving in his "Beobachtungen" conveys, though in a rough manner, its characteristic features; the disease, which occurred in the stomach, was however described by him as scirrhus of that organ. But to return to Dr. Walshe's case:

"The morbid matter occupied about one third of the posterior surface of the organ, terminating at about half an inch from the pylorus, and formed an irregular globular elevation somewhat more than half an inch high (exclusive of the coats of the stomach), two broad, and two and a half wide. Over by far the greater part of its surface, which was irregularly mammillated, and of a greenish yellow tint, the morbid matter was uncovered, and exhibited its characteristic alveolar and jelly-like aspect. But on the confines these characters were only seen in some spots, where destruction of the investing mucous membrane had taken place: this destruction seemed to consist in a simple wearing out from gradual attenuation; there was no hardening or elevation of the borders of the small perforations thus produced, nor had they the appearance of ordinary ulcers. Surrounding this mass, on its cardiac border, was an elevated flabby formation, more than half an inch high, and three lines thick in some places, with the feel of a piece of lung condensed by simple pressure. On making a section of this production, the colloid tumour and the coats of the stomach, the following were found to be their condition and mode of relation. The peritoneum was everywhere traceable, and in some parts considerably thickened; the muscular tunic, of a pale yellow-green tint, varied from $\frac{1}{8}$th to $\frac{1}{4}$th of an inch in thickness; the major part of the colloid mass lay on the gastric surface of the cellulo-fibrous membrane, to which it adhered closely; in one or two points of its periphery the diseased structure penetrated, as it were, through this membrane and the muscular coat down to the peritoneum, along the inner surface of which it then spread for about half an inch. Where the mucous membrane existed entire over the colloid structure, it could be dissected from off the latter without injuring the cancerous cells; a fact showing that the morbid matter was here developed between the cellulo-fibrous and the mucous tunics. But the colloid alveoli also extended, gradually decreasing in closeness and number, into the elevated production already referred to: this seemed itself to be nothing more than a hypertrophous state of a fold of the mucous coat, accompanied with flabby induration; it had neither the structure nor the consistence of any form of indurated carcinoma, and its gastric surface was perfectly sound and apparently healthy. Hence it appears that the mucous membrane, previously rendered hypertrophous, may, as well as the cellulo-fibrous and mucous tunics, become the nidus of the diseased formation." (pp. 600-1.)

Under the head of Physiology are examined some of the phenomena influencing and attending the evolution of carcinoma; the circumstances of its origin, growth, and decay. The different species are, contrary to the plan adopted with their anatomy, considered together, "in order to avoid the repetitions which, on account of the similarity of their physiological processes, a separate examination of each would unavoidably entail."

Dr. Walshe subjects to a very searching criticism the principal views which have been broached in regard to the origin of carcinoma, by Pouteau, Abernethy, C. Wenzel, Broussais, Breschet, Ferrus, Andral, Hodgkin, Cruveilhier, Carswell, and he concludes as follows:

"From these investigations, however, an important fact derives: the microscopical elements of cancerous growths and their mode of propagation are identical with those not only of benignant tumours, but of the normal embryonic tissues. But we cannot agree with Müller in his inference, that this identity proves carcinoma not to be a heterologous formation. As it appears to us, such identity simply shows that the heterologous character, visible and palpable as it is, is produced not by the nature, but by the mode of combination and arrangement of the ultimate physical elements of the diseased growth. As well might it be contended that calomel and corrosive sublimate are not heterologous to each other, because they are both composed of atoms of chlorine and mercury." (p. 610-11.)

It may be well to remark, Dr. Walshe further says, that there is no real similarity between Müller's doctrine and the cystic theory of Dr. Hodgkin, as in the one case the phenomena are microscopical, and in the other putatively traceable with the unassisted eye.

Under the head of Growth, Dr. Walshe gives a short abstract of Schwann's theory regarding the development of the normal embryonic tissues, and Professor Müller's application of it to the explanation of the growth of carcinoma, and then makes the following comments:

"It will be observed that the origin of the cytoblasts is in this theory the unsolved problem; until this be decided, whatever light Müller's researches may have thrown on the mode of increase of these products, they have by no means cleared up the history of their origin. The only probable method of discovering the cause and mode of their primitive production, the physical change directly preceding it, namely, a minute scrutiny of the state of the vessels leading to the molecular seat of thedisease, has not been pursued by this observer." (p. 610.)

The following remarks on Melanosis are worthy of notice:

"Carcinoma occasionally presents a black tinge, either from abundant punctuation, infiltration, or deposition in masses of *melanic matter;* and an attempt has been made by some authors, led by this accidental impregnation into a belief that melanosis constitutes a separate malignant formation, to class it as a species of cancer. With the holders of this notion we cannot, for the following reasons, agree: 1. Pure melanic pigment, no matter in what abundance it be accumulated, produces no other effects than those dependent on the mechanical obstruction caused by the mass it constitutes. 2. When melanotic tumours produce the local and general symptoms of cancerous growths, they are found to be composed either of encephaloid or scirrhus (more especially the former), impregnated with black matter. 3. Neither the local nor general symptoms of carcinoma are modified in cases in which melanic matter is found to pervade it. 4. Melanic matter is incapable of forming a tissue, being an unorganized fluid into which vessels have never been observed to penetrate. It has been remarked, that when one cancerous growth is tinged with black matter, any others in the body are almost invariably found similarly discoloured: this fact, to which much importance has been attached, simply proves that there is a tendency in the system to the production of melanic matter. Again, Cruveilhier lays much stress on the circumstance that "melanic cancer" is rarely solitary; this is the necessary result of the fact, that black or bistre coloured impregnation much more frequently complicates encephaloid than the other species of carcinoma. The French pathologist has not thought of proving numerically that such multiplicity of growth is less frequent when the encephaloid is of normal than of abnormal colour." (p. 614.)

Under the head of pathology, the cause of the greater tendency of some organs than of others to cancerous deposition is enquired into, and the conclusion drawn is, that it is unknown. The anatomical course of the disease is next traced, then the condition of the solids and fluids of subjects dying with cancerous disease glanced at. The *etiology* is next very fully considered in all its bearings; and lastly, the duration, frequency, symptoms, diagnosis, and prognosis are each treated of in succession.

The disease may, in its material manifestation, be confined to a single organ from the outset to the fatal termination; or it originates in a single organ or tissue, whence it seems to spread, as from a centre, to a multitude of parts: the latter are said to be affected with *secondary* cancer. How does transmission of the disease to distant parts take place? This, answers Dr. W., seems only likely to be through the lympathic or venous system.

In regard to the doctrine of venous transmission, Dr. Walshe remarks that some apparently serious objections may be raised against it. He says, "If such, it may be urged, be the mechanism of the production of carcinoma in distant organs, why is such production not a constant phenomenon? why should cancerous disease ever terminate the existence of individuals without having manifested itself *materially* in more than a single spot? A satisfactory answer cannot, in the present state of science, be easily found. .... Another objection to this doctrine may appear in the multitude and large size of secondary formations; it is physically impossible, it may be alleged, that these should have been the produce of absorption from a mass, itself smaller than many among them, and which at no period of its growth suffered apparent diminution of bulk. But it is not meant in the remotest degree (and this is the answer to those who object to the origin of secondary carcinoma in absorption on the score of its consistence), that consecutive formations are composed of the actual substance of the primary mass, though even this does not seem, from the evidence of infinitely rare cases, to be impossible; the micrography of cancer shows that the translation and deposition of a few cells only from the original nidus might lead to the development of the largest mass: each cell is in itself *the possible embryo of a tumour.* There is yet another fact, apparently subversive of the notion of venous transmission: the detritus and ichor of carcinoma have been injected into the veins of animals, but no development of cancerous structure has resulted from the experiment. Granted; but this simply proves—and it does so, perhaps, as conclusively as any other adducible argument—the absolute necessity of predisposition for the production of the disease; without this, even its material constituent manifests itself only as an irritative agent." (p. 620.)

After a further critical enquiry, distinguished by great acumen, into the true mechanism of secondary cancerous development, Dr. Walshe says,

"The reader has probably felt inclined to ask, where is the proof that, in the cases of general infection adverted to, the growths which we have considered to be developed consecutively to another or others, really possess such relation thereto? Is it not quite as likely that, in some cases at least, the presence of heterologous growths in numerous organs may depend on their simultaneous development. Unquestionably such is the truth in a greater share of instances than the language of reporters of cases would lead us to suppose." (p. 622.)

*Etiology.* This part is very ably treated of and illustrated by much statistical information. The two following tables we extract as illustrative of the influence which age exerts as a predisposing cause.

*Table, showing the absolute mortality from Carcinoma in both sexes and at all ages.*

| Age. | Males. | Females. | Both Sexes. |
|---|---|---|---|
| 1 month | | | |
| 2 months ... | ... | 1 | 1 |
| 3 and under 6 | ... | 1 | 1 |
| 6 " 9 | | | |
| 9 " 12 | | | |
| 1 year ... | 2 | 1 | 3 |
| 2 years ... | 1 | 4 | 5 |
| 3 " ... | ... | 1 | 1 |
| 4 " ... | ... | 1 | 1 |
| 5 and under 10 | 3 | 2 | 5 |
| 10 " 15 | 1 | 4 | 5 |
| 15 " 20 | 3 | 5 | 8 |
| 20 " 25 | 4 | 2 | 6 |
| 25 " 30 | 1 | 13 | 14 |
| 30 " 35 | 6 | 23 | 29 |
| 35 " 40 | 15 | 43 | 58 |
| 40 " 45 | 19 | 77 | 96 |
| 45 " 50 | 23 | 98 | 121 |
| 50 " 55 | 34 | 130 | 164 |
| 55 " 60 | 35 | 120 | 155 |
| 60 " 65 | 44 | 110 | 154 |
| 65 " 70 | 45 | 88 | 133 |
| 70 " 75 | 35 | 69 | 104 |
| 75 " 80 | 30 | 49 | 79 |
| 80 " 85 | 16 | 28 | 44 |
| 85 " 90 | 1 | 8 | 9 |
| 90 " 95 | 2 | 1 | 3 |
| 95 and upwards ... | 1 | ... | 1 |
| Totals. | 321 | 879 | 1200 |

In regard to this table, Dr. Walshe says (and the remarks indicate the comprehensiveness and caution of the accomplished medical statist,)

"We learn the proportional number of individuals dying from carcinoma at each specified period of life; but were we to conclude that these numbers represent the relative tendency to the disease at different ages, we should fall into a very serious error. It appears, for example, that 250 females die of cancer between the ages of 50 and 60, while 118 perish between those of 70 and 80; but, if the total number of females living of the former age were double that of those alive of the latter, proportional mortality at both decennial periods of life would be as nearly as possible equal, instead of being in one of them double that of the other. In a word, in order to ascertain whether particular ages exercise any influence on the development of the disease, we must compare the absolute mortality with the total number living at each age. This we have been enabled to do from the population estimates of Mr. Rickman, and present the reader with the result in the following

*Table, showing the proportion of Deaths from Cancer in* 1,000 *living of each sex at the different Ages.*

| Ages. | Males. | Females. | Mean. |
|---|---|---|---|
| Under ...... 5 | ·006 | ·017 | ·012 |
| 5 and under 10 | ·007 | ·004 | ·006 |
| 10 " 15 | ·002 | ·010 | ·006 |
| 15 " 20 | ·009 | ·017 | ·013 |
| 20 " 30 | ·010 | ·024 | ·017 |
| 30 " 40 | ·058 | ·152 | ·105 |
| 40 " 50 | ·140 | ·983 | ·561 |
| 50 " 60 | ·290 | 1·066 | ·678 |
| 60 " 70 | ·636 | 1·192 | ·919 |
| 70 " 80 | ·935 | 1·421 | 1·178 |
| 80 " 100 | 1·207 | ·973 | 1·089 |
| All ages ......... | ·103 | ·245 | ·174 |

"A glance at this table," Dr. Walshe remarks, "will suffice to show the inaccuracy of the commonly-received opinion, that the tendency to cancer reaches its maximum between the 35th and 50th years. The mortality, on the contrary, goes on steadily increasing with each succeeding decade until the 80th year; after that period the results are, from causes which it is needless to enumerate, liable to slight error, and may therefore be omitted in the general survey." (p. 625.)

In farther illustration of this interesting subject, a diagram is given, reduced from the preceding table, *showing the relative mortality from cancer at different ages in both sexes;* for which, and many other discussions of surpassing interest, we must refer the reader to the original.

*Therapeia.* The cure of cancer! the philosopher's stone! the catholicon! Such things Dr. Walshe has carefully shut the doors of his imagination against, and encounters the subject with soberness and truth.

"The following propositions," says he, "appear to embrace such facts as are established respecting the curability of carcinoma, by art. 1. There is no existing evidence to show that carcinoma of an internal organ has ever been cured. 2. Cancer has never been removed by medicinal agents alone. 3. Morbid productions possessing the anatomical and pathological character of scirrhus have been removed with the knife, and no return of the disease been observed for a number of years, and occasionally during the entire course of a life, prolonged beyond the expectation of life in healthy individuals at the period of removal. 4. Although it cannot be doubted that many of the tumours referred to by M. Recamier were not really composed of that tissue, yet it seems impossible to affirm this of all of them: it would follow then that carcinoma has in rare instances been cured by compression, aided by other external and internal remedies. 5. The probabilities of effecting a cure vary with the species of cancer. There are few authentic instances on record of permanent recovery after the ablation ef encephaloid; successful removal of scirrhus is of more frequent occurrence, and the tendency of colloid to reproduction, although Velpeau states it to be extreme, is probably lower than of the other varieties. 6. The earlier the morbid mass is removed, the stronger are the chances of ultimate recovery: Scarpa's three successful operations were performed within the first three months; of twenty-seven scirrhi removed by Flajani within the first month of their formation, two only returned. 7. Encysted cancer, provided the cyst be removed, is less liable to return than non-encysted. 8. The chances of success diminish out of all proportion with each repetition of the operation. 9. It has been stated that the removal of cancer in some parts is more prone to be attended with relapse than in others: mammary cancer is instanced as an example of the former, osseous of the latter. We are disposed to acquiesce in this notion, but it requires to be proved numerically; and the fact that under the term osteo-sarcoma have been included growths in bone of almost every description, fibrous, cartilaginous, &c., as well as the truly cancerous, renders it exceedingly probable, if not certain, that the supposed immunity of bone in this respect has been exaggerated. 10. It is indubitable that local, and probably distant, reproduction frequently depends on some of the diseased growth being left behind. ........ The inference is that, were ablation perfect, recoveries would be more common." (p. 640.)

"Finally," says Dr. Walshe, in the penultimate paragraph of the first part of his memoir, "the following proposition directly flows from numerous facts stated in the preceding pages: a cancerous tumour, under all circumstances, even should it remain single and stationary for years, is but the local evidence of a general vitiation of the system. But, it may be enquired, is not this a contradiction? how is it possible, that if the organism at large be in a state of disease, a single spot shall alone manifest its presence? We confess our inability to explain away this difficulty; but neither the fact of its existence, nor its inexplicability shake the solidity of the opinion advocated. All pathologists who have carefully investigated the history of the tuberculous affection admit that local agencies, of whatever kind they may be, can never cause the production of a particle of tubercle, that constitutional derangement must lead the way. Now here we have precisely the same difficulty to contend with; a knot of tubercles may exist for years in the summit of a lung, and every other organ remain free to the last from the disease. Why this is so, we know not: these are the mysteries of pathology, and they are mysteries which it would, in the existing state of the science, be idle to attempt to penetrate." (p. 650.)

Our limited space forbids us accompanying Dr. Walshe in the second part of his memoir, of which indeed any abstract of ours would give but a very insufficient idea. We can recommend it to the practitioner as a most valuable source of reference. It is a cyclopædia of the accumulated experience of practitioners on cancer in various parts of the body. Were we to direct attention to any one part of the subject in particular, it would be to the article on cancer of the meninges.

It will be seen that the preceding notice of Dr. Walshe's memoir can scarcely be looked upon either as an analytical or critical review, because to have attempted making it so would have been supererogatory, as the memoir itself is not only a dissertation on the nature and cure of cancer, but a masterly analytical and critical review of the whole bearings of the subject. We have therefore principally confined ourselves to the quotation of such passages as appeared to us likely to interest our readers and to give them an idea of the execution of the whole original, the careful study of which we most cordially recommend.

